# Job strain and loss of healthy life years between ages 50 and 75 by sex and occupational position: analyses of 64 934 individuals from four prospective cohort studies

**DOI:** 10.1136/oemed-2017-104644

**Published:** 2018-05-07

**Authors:** Linda L Magnusson Hanson, Hugo Westerlund, Holendro S Chungkham, Jussi Vahtera, Naja H Rod, Kristina Alexanderson, Marcel Goldberg, Mika Kivimäki, Sari Stenholm, Loretta G Platts, Marie Zins, Jenny Head

**Affiliations:** 1 Stress Research Institute, Stockholm University, Stockholm, Sweden; 2 Department of Clinical Neuroscience, Division of Insurance Medicine, Karolinska Institutet, Stockholm, Sweden; 3 Indian Statistical Institute, North-East Centre, Tezpur University, Tezpur, India; 4 Department of Public Health, University of Turku and Turku University Hospital, Turku, Finland; 5 Department of Public Health, Copenhagen University, Copenhagen, Denmark; 6 Population-based Epidemiologic Cohorts Unit-UMS 011, Inserm, Villejuif, France; 7 Aging and Chronic Diseases, Epidemiological and Public Health Approaches, Inserm, Villejuif, France; 8 Department of Epidemiology and Public Health, University College London, London, UK; 9 Clinicum, Faculty of Medicine, University of Helsinki, Helsinki, Finland; 10 School of Health Sciences, University of Tampere, Tampere, Finland

**Keywords:** epidemiology, stress, longitudinal studies, organisation of work, workload

## Abstract

**Objectives:**

Poor psychosocial working conditions increase the likelihood of various types of morbidity and may substantially limit quality of life and possibilities to remain in paid work. To date, however, no studies to our knowledge have quantified the extent to which poor psychosocial working conditions reduce healthy or chronic disease-free life expectancy, which was the focus of this study.

**Methods:**

Data were derived from four cohorts with repeat data: the Finnish Public Sector Study (Finland), GAZEL (France), the Swedish Longitudinal Occupational Survey of Health (Sweden) and Whitehall II (UK). Healthy (in good self-rated health) life expectancy (HLE) and chronic disease-free (free from cardiovascular disease, cancer, respiratory disease and diabetes) life expectancy (CDFLE) was calculated from age 50 to 75 based on 64 394 individuals with data on job strain (high demands in combination with low control) at baseline and health at baseline and follow-up.

**Results:**

Multistate life table models showed that job strain was consistently related to shorter HLE (overall 1.7 years difference). The difference in HLE was more pronounced among men (2.0 years compared with 1.5 years for women) and participants in lower occupational positions (2.5 years among low-grade men compared with 1.7 years among high-grade men). Similar differences in HLE, although smaller, were observed among those in intermediate or high occupational positions. Job strain was additionally associated with shorter CDFLE, although this association was weaker and somewhat inconsistent.

**Conclusions:**

These findings suggest that individuals with job strain have a shorter health expectancy compared with those without job strain.

## Introduction

Life expectancy continues to rise globally. However, health expectancy such as disability-free life expectancy or healthy life expectancy, which according to WHO refers to the “average number of years that a person can expect to live in ‘full health’ by taking into account years lived in less than full health due to disease and/or injury”, has not risen to the same extent.^1^ This suggests that some people live longer in suboptimal health.

In line with increases in life expectancy, several European governments strive to extend working lives into higher ages. Suboptimal health may, however, limit possibilities for labour market participation at older ages. Extended working lives also means that people are exposed to their work environment for a longer period, which could have both positive and negative effects on health and health functioning.^2–4^ There is a large body of evidence suggesting that a poor psychosocial work environment is associated with increased risks of various health problems. For example, high job demands and low job control, or the combination thereof (referred to as job strain), have been found to predict coronary heart disease,^5^ ischaemic stroke,^6^ diabetes,^7^ musculoskeletal disorders^8^ and mental health.^9^ However, no previous study has to our knowledge assessed the potential contribution of psychosocial working conditions to more comprehensive measures of mortality, physical and mental health such as health expectancy.^10^

In the present study, we examine the extent to which job strain is associated with healthy life expectancy (HLE) and chronic disease-free life expectancy (CDFLE) among women and men from Finland, France, Sweden and the UK between the ages of 50 and 75 years.

## Methods

Data from four prospective occupational cohort studies in Finland (Finnish Public Sector Study), France (GAZEL), Sweden (Swedish Longitudinal Occupational Survey of Health) and the UK (Whitehall II), from in total 64 934 individuals, were used. As our aim was to study the influence of job strain in later working life, participants were included at the time of the first observation with data on health when they were aged 50 years or older and were followed until age 75 at the longest (a maximum follow-up time of 26 years), for comparability between the cohorts. The included cohorts are briefly described below, and the number of individuals in each of the cohorts and follow-up time according to age at inclusion are presented in online supplementary table 1. More details of the cohorts have been presented elsewhere.^11–17^ The study was conducted in accordance with the Declaration of Helsinki of the World Medical Association. Ethical approval was given for each cohort in each country from relevant ethical committees/boards. Informed consent was obtained for all participants.

10.1136/oemed-2017-104644.supp1 Supplementary data

### Cohorts

Data from Finland were obtained from the Finnish Public Sector Study (FPS). The study, established in 1997/1998, comprises all 151 901 employees with a ≥6-month job contract in any year from 1991/2000 to 2005 in 10 towns and five hospital districts in Finland.^12^ Survey data have been collected by repeated surveys at 4-year intervals on all 103 866 cohort members, who were at work in the participating organisations during the surveys in the years 1997/1998, 2000/2001, 2004/2005, 2008/2009 and/or 2012/2013. Follow-up survey data of the respondents who had retired or left the organisations were collected in 2005, 2009 and 2013. Of those, 84 848 participants responded at least once (response rate 82%). In this study, data from five waves were used. People in paid work who fulfilled the inclusion criteria were followed up to 16 years (mean follow-up time 6.8 years; online supplementary table 1). In total, 36 317 people also had data on self-rated health and on disease.

The French data were derived from the GAZEL Cohort Study, set up in 1989 among workers at Électricité de France-Gaz de France (EDF-GDF), the French national utility company. At inception in 1989, GAZEL included 20 625 volunteers (15 011 men and 5614 women) working at EDF-GDF aged 35–50 years.^13^ For the analysis, data from annual waves of GAZEL with almost 75% response rate every year were used. In this study, job strain was recorded for people in paid work either in 1997 or 1999. Participants who fulfilled the inclusion criteria were followed for up to 17 years (mean follow-up time 13.8 years; online supplementary table 1). In total, 11 302 people additionally had data on self-rated health and chronic disease.

The data for Sweden came from five waves of the Swedish Longitudinal Occupational Survey of Health (SLOSH), which is a longitudinal follow-up study of respondents to the biennial cross-sectional Swedish Work Environment Survey (SWES), which in turn targets a random stratified sample of gainfully employed Swedish residents aged 16–64 years.^15 16^ The first wave of SLOSH in 2006 comprised all respondents from SWES 2003. At wave 2 in 2008, the sample was increased by adding the respondents from SWES 2005. This resulted in a total sample of 18 917 persons originally representative of the working population in Sweden in 2003 and 2005, who were then re-surveyed in 2010, 2012 and 2014.^16^ In 2010, a geographical subsample of participants from SWES 2007 was also contacted whereas all participants of SWES 2003–2011 were invited to respond to questionnaires 2014. The analytic sample in the present study comprised participants who had responded to at least one wave (65% of all SWES participants). In this study, people in paid work who fulfilled the inclusion criteria were followed up to 8 years (mean follow-up time 6 years; online supplementary table 1). In total, 8194 people also had data on self-rated health while 8070 people had data on chronic diseases.

The data from UK came from the WHII, a prospective cohort of British civil servants established in 1985–1988 when 10 308 participants aged 35–55 years were recruited to the study.^17^ Since then, follow-up surveys have taken place approximately every 2 to 3 years with response proportions ranging between 61% and 79%. In this study, job strain was recorded for people in paid work in 1985–1988. Participants who fulfilled the inclusion criteria were followed up to 28 years (mean follow-up time 15.2 years; online supplementary table 1). In total, 9121 people also had data on self-rated health and 9143 had data on chronic disease.

### Job strain

A harmonised measure of job strain was used in the present study.^18^ The definition of job strain was based on self-report questionnaires in all studies assessing job demands and job control. Complete scales based on Karasek’s job demand-control model^19^ were used in the GAZEL study (based on the Job Content Questionnaire) and SLOSH (based on the Demand Control Questionnaire), while partial scales for demands were used in the Finnish Public Sector Study (based on the Job Content Questionnaire) and the WHII (based on the Job Content Questionnaire) as shown in online supplementary table 2. The included partial scales have been shown to have a high correlation with the complete scales and is expected to capture the same underlying construct.^18^ Based on the Likert scales response formats, we calculated mean response scores for demands and control, respectively, for each participant. Persons scoring above the study-specific median on the demand scale and below the study-specific median on the control scale were defined as exposed to job strain.^6^ All other combinations of demands and control were defined as no job strain in line with most commonly used categorisation. Previous work have demonstrated a good correspondence between job strain measures based on the complete original scales and between job strain measures based on least one complete scale,^18^ as was the case in the present study.

10.1136/oemed-2017-104644.supp2 Supplementary data

### Outcome measures

**Figure 1:**
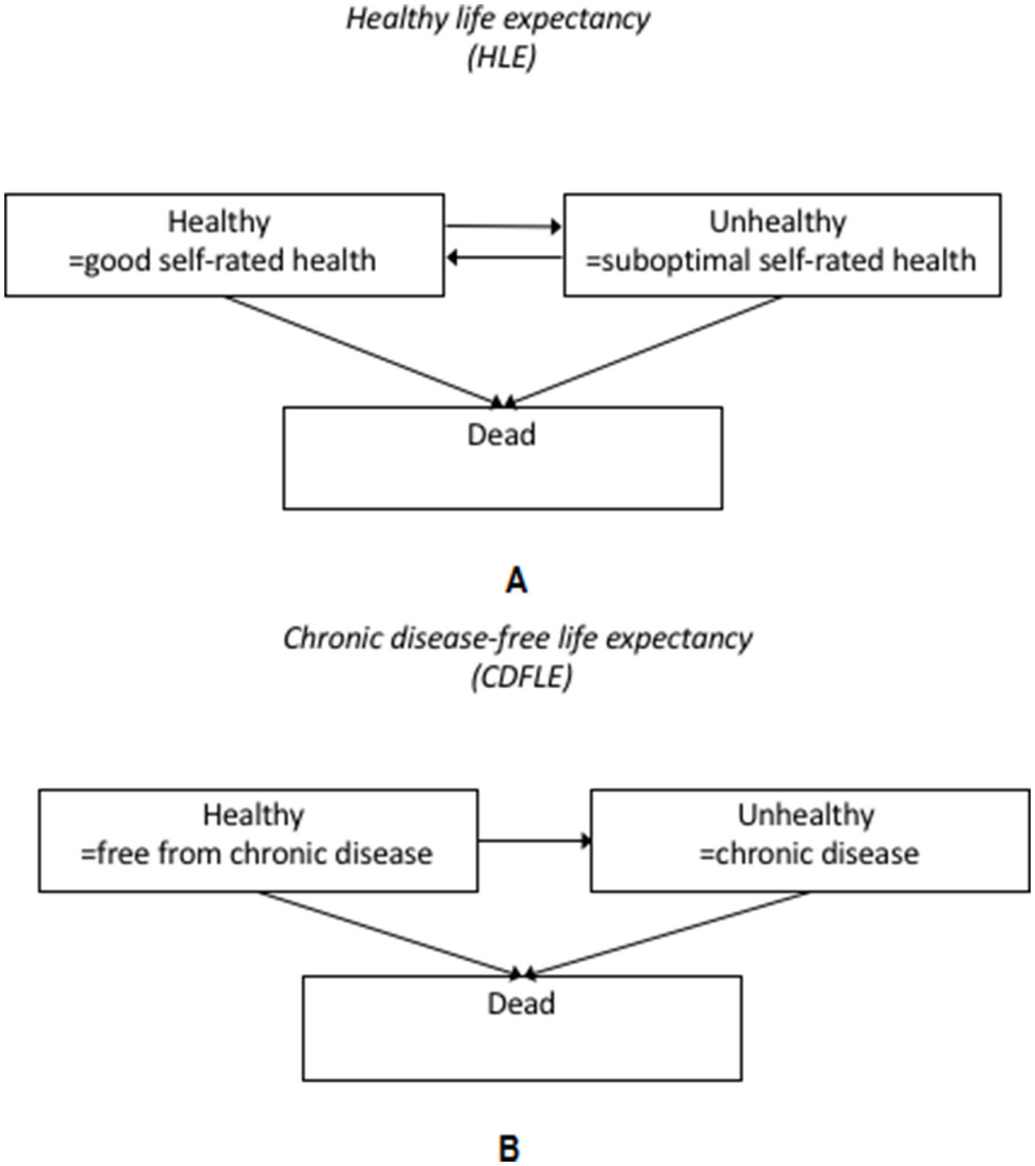
(A) Definition of the healthy life expectancy (HLE) measure. The figure illustrates the three possible health states considered, based on self-perceived health status and mortality data, and the four possible transitions between the different states. (B) Definition of the chronic disease-free life expectancy (CDFLE) measure. The figure illustrates the three possible health states considered, based on data on chronic diseases (including heart disease, stroke, diabetes, lung disease and cancer) from self-reports or registers and mortality, and the three possible transitions between the different states.

All participants were followed up each wave with regard to health and disease, whether or not participants remained in paid work or not. Mortality was also ascertained from linked register data with follow-up censored on 31 December of the year in which data collection on health and disease last took place for each study cohort. Information on mortality was used to estimate partial life expectancy between the ages of 50 and 75. Furthermore, three health states were defined: healthy, unhealthy and dead, in order to estimate partial health expectancies between the ages of 50 and 75 in terms of healthy life expectancy and chronic disease-free life expectancy. The measure of HLE was based on mortality data in combination with self-rated health. Responses were categorised into good and suboptimal health. There were some differences between the cohorts regarding the specific items used for ratings of self-rated health. GAZEL participants indicated their health state from very good to very bad on an eight-point scale. In line with previous research,^4^ the four top response options were coded as good health. In WHII, SLOSH and the Finnish Public Sector Study, the question used a five-point response format, ranging from Excellent/Very good to Very poor. For the Finnish Public Sector Study and SLOSH, the top two response options (Quite good/Good, Very good) were grouped and categorised as ‘good health’,^20^ and in WHII, the top three response options (Good, Very good, Excellent) were categorised as ‘good health’ for comparability. More details of the questions and response options are provided in online supplementary table 2. The three different health states considered for HLE are illustrated in figure 1A, along with the possible transitions between the different states.

For CDFLE, mortality and chronic disease status were used to define the three different health states as basis for the analyses (figure 1B). The presence of a chronic diseases was ascertained for GAZEL, the Finnish Public Sector Study and SLOSH by self-reports (online supplementary table 2). The chronic diseases included (1) heart disease (heart attack, coronary heart disease, angina, congestive heart failure or other heart problems), (2) stroke (stroke or transient ischaemic attack), (3) chronic lung disease (chronic bronchitis or emphysema or asthma), (4) cancer (cancer or a malignant tumour of any kind except skin cancer) and (5) diabetes (diabetes or high blood sugar). For WHII, a combination of self-report and cancer register data was used. Individuals were defined as having a chronic disease if they reported one or more of the above conditions. The presence of chronic disease at baseline (first observation included in analysis) included any chronic conditions reported before or at the age of 50 from available information on respondents. However, to ensure comparability across studies, the data for Sweden on chronic conditions came from the 2008–2014 waves, as the 2006 wave did not collect information on all chronic conditions.

### Demographic characteristics

Sex was determined in all cohorts from self-reports or registers. In the Finnish Public Sector Study, occupational position was obtained from employer records and categorised into three groups: higher (eg, teachers, physicians), intermediate (eg, registered nurses, technicians) and lower (eg, cleaners, maintenance workers). In GAZEL, occupational position was obtained from administrative records and analogously categorised into three groups: higher (eg, managers), intermediate (eg, administrative associate professionals and technicians) and lower (eg, manual worker and clerks). In SLOSH, occupational position was based on self-reported job title and also categorised into three groups: higher (eg, upper-level executives, professionals and other higher non-manual employees), intermediate (eg, assistant and intermediate non-manual employees) and lower (eg, manual workers). However, as this variable has not been coded for the 2006 wave of SLOSH, the socioeconomic indicator in that year was carried forward from the classification in the 2003 SWES. In WHII, occupational position was measured by civil service employment grade, which was closely related to income and categorised into three groups: high administrative (civil service unified grades 1–7 or equivalent), professional and executive, and clerical and support staff (eg, messengers, porters, telephonists, typists).^17 21^ The three categories were labelled high, middle and low grade occupational position across the cohorts.

### Statistical analyses

Multistate life table models were used to estimate life expectancies, HLE and CDFLE between the ages of 50 and 75. These analyses were conducted separately for HLE and CDFLE. For HLE based on self-rated health, there were four possible transitions between the health states: healthy to unhealthy (onset), unhealthy to healthy (recovery), healthy to dead and unhealthy to dead. For CDFLE based on chronic diseases, there were only three possible transitions as recovery was not allowed (figure 1A,B). For each cohort, age-specific transition probabilities between the health states by sex, occupational position (low, middle or high) and job strain were estimated from multinomial logistic models with age (in years), sex and occupational position as covariates. These estimated age-specific transition probabilities were used as inputs for calculation of life expectancy and health expectancies. For each cohort, individual trajectories of health and mortality from age 50 to age 75 for a simulated cohort of 100 000 persons were generated by a microsimulation approach,^22^ with distributions of covariates at baseline based on the observed age-study-specific prevalence of sex, occupational position and job strain. Life expectancies, HLE and CDFLE from age 50 to 75 were calculated as the average from these trajectories for each occupational position and sex. The results for CDFLE were presented for all individuals but also specifically for people without chronic disease at baseline among whom exposure to job strain is more likely to have preceded disease development. The covariate selection was based on a priori knowledge, statistical criteria and comparability between the cohorts. According to previous research and differences in disease risk, the analyses were stratified by sex. We also investigated multiplicative interaction with sex and occupational position, by including interaction terms between job strain, sex and occupational position in the multinomial regression models. Computation of standard errors and 95% CIs (25th and 95th percentiles) for these multistate life table estimates were performed using a bootstrap method with 500 replicates for the whole analysis process (multinomial analysis and simulation steps). All analyses were conducted in SAS V.9.2 using the SPACE (Stochastic Population Analysis of Complex Events) program (http://www.cdc.gov/nchs/data_access/space.htm). This program specifically developed for large-scale complex surveys uses a stochastic (ie, microsimulation) approach to estimate measures of life expectancy and health expectancies. The cohort-specific results were pooled through fixed-effects meta-analysis using R. To test the robustness of the results, we also repeated the analyses with job strain (high demands and low control) compared with low strain (low demands and high control), as opposed to all other combinations of demand and control.

## Results

### Population characteristics

The prevalence of sociodemographic and health characteristics at baseline in the respective cohorts are presented in table 1. Most people were in the age range 50–59 at baseline and about 14%–20% reported job strain. At baseline, the prevalence of suboptimal self-rated health ranged from 20% in the French sample (GAZEL) to 36% in the Finnish sample (Finnish Public Sector Study) (table 1). The prevalence of self-reported chronic disease ranged from 21% in SLOSH to 27% in the Finnish Public Sector Study.

**Table 1:** Prevalence (%) of sociodemographic and health characteristics at baseline

	Finnish Public Sector Study (Finland)	GAZEL (France)	Swedish Longitudinal Occupational Survey of Health (Sweden)	Whitehall II (UK)
Sample size at baseline	36 317	11 302	8194	9121
Sex (%)				
Male	21	73	46	68
Female	79	27	54	32
Age group (%)				
50–54	72	91	42	84
55–59	23	9	24	14
60–64	4	0	24	2
65–69	0	–	10	0
70–74	–	–	0	0
Occupational position (%)				
High grade	31	28	19	31
Middle grade	48	57	45	48
Low grade	21	15	36	21
Job strain (%)				
No job strain	80	86	80	86
Job strain	20	14	20	14
Self-rated health (%)				
Good	64	80	78	79
Suboptimal	36	20	22	21
Chronic disease*	(n=36 317)	(n=11 302)	(n=8070)	(n=9143)
No	73	76	79	76
Yes	27	24	21	24

*Presence of chronic disease includes illness reported at or before baseline.

### Results of the multinomial regression models

For all cohorts, there was an increased risk of poor self-reported health (or mortality) from a state of good health for people with job strain. Correspondingly, the likelihood of recovery from poor health was lower for people with job strain (see online supplementary table 3). Similarly, job strain was associated with increased likelihood of transitioning to chronic disease from a state free of chronic diseases (see online supplementary table 4). However, the estimates of mortality risk from an unhealthy state showed a less consistent pattern by job strain (online supplementary tables 3 and 4). There was a statistically significant interaction between job strain, sex and occupational position in the multinomial regression models in three out of the four cohorts (FPS, GAZEL and WHII, P=0.00–0.04) when analysing transition probabilities between good self-rated health, suboptimal self-rated health and death. In analyses of transition probabilities between the states of free of chronic disease, chronic disease and death, there was a statistically significant interaction between job strain, sex and occupational position in the multinomial regression models in the FPS and WHII cohorts (P=0.00–0.01).

### Healthy life expectancy

The results of the multistate models showed that people with job strain had shorter HLE from age 50 to 75 than those without job strain. The results indicated that those without job strain lived 19.5 (18.3–20.8) years with good health, whereas those with job strain lived on average 17.8 (16.3–19.3) years in good health, when estimates were pooled across cohorts, sex and occupational position, suggesting a 1.7 years shorter HLE among those with job strain.

#### HLE stratified by sex

The estimates of HLE differed most notably between men and women. The pooled HLE between the ages of 50 and 75 for men and women, by job strain, are shown in table 2. The pooled HLEs ranged from 17.4 years in men with job strain to 19.7 years in women without job strain. The difference in HLE between people with and without job strain was 1.5 years in female employees and 2.0 years in male employees. The corresponding proportions of life spent in good health ranged from 72% among men with job strain to 80% among men without job strain (online supplementary table 5). The results of the multistate models stratified by sex also showed that there were no significant differences in overall life expectancy between the job strain and no job strain groups (online supplementary table 5).

**Table 2:** Healthy life expectancy between the ages of 50 and 75 by job strain, sex and occupational position

	Men		Women	
	n	Healthy life expectancy (95% CI)*	n	Healthy life expectancy (95% CI)*
All	25 731		39 203	
No job strain	21 683	19.4 (19.2 to 19.6)	31 627	19.7 (19.5 to 19.9)
Job strain	4048	17.4 (17.0 to 17.8)	7576	18.1 (17.9 to 18.4)
High grade				
No job strain	7839	20.9 (20.5 to 21.2)	6993	21.1 (20.8 to 21.4)
Job strain	1448	19.2 (18.5 to 19.9)	2553	20.0 (19.4 to 20.5)
Middle grade				
No job strain	9097	19.7 (19.4 to 20.0)	17 495	20.2 (20.0 to 20.5)
Job strain	1629	17.9 (17.3 to 18.6)	3785	18.6 (18.2 to 19.0)
Low grade				
No job strain	4747	17.5 (17.1 to 17.9)	7139	17.7 (17.3 to 18.1)
Job strain	971	15.0 (14.4 to 15.7)	1238	15.9 (15.4 to 16.3)

*Estimated life years spent in good self-rated health between ages of 50 and 75.

#### HLE additionally stratified by occupational position

**Figure 2:**
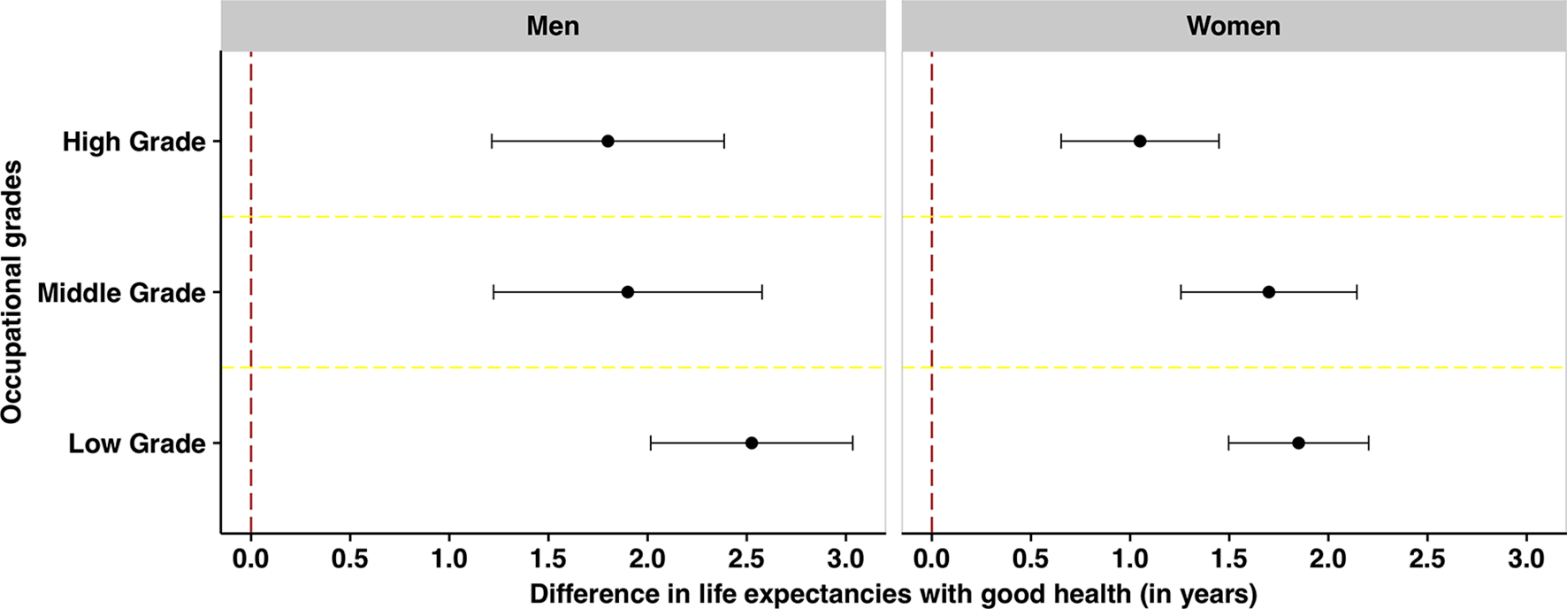
Differences (in years) in healthy life expectancies (HLEs) between people without job strain and with job strain by sex and occupational position. The figure illustrates how many years shorter HLE is predicted to be for people with job strain compared with people without job strain within each stratum.

The estimates of HLE also differed to some extent between occupational grades (table 2). The differences in healthy life years by sex and occupational position are also shown in figure 2, which illustrates that those with job strain had between 1.1 and 2.5 years shorter HLE than those without job strain. Job strain was primarily associated with fewer years with good health for men and women in low-grade occupational positions. There was a consistent pattern across cohorts, although the differences in healthy life years differed slightly between cohorts (online supplementary table 9a,b).

### Chronic disease-free life expectancy

Among the full sample with data on chronic disease (n=64 832), the multistate models indicated that those with job strain had overall 1.2 years shorter CDFLE than those without job strain. The corresponding stratified results are presented in online supplementary tables 6, 10a,b and online supplementary figure 1.

The differences in CDFLE were, however, less pronounced among people without chronic disease at baseline (n=48 425). Overall, those with job strain had 0.7 years shorter CDFLE than those without job strain.

#### CDFLE stratified by sex

Men with job strain and without chronic disease at baseline had 0.8 years shorter CDFLE, and women with job strain without chronic disease at baseline had 0.6 years shorter CDFLE than those without job strain (table 3). The CDFLEs in this group were generally shorter than the HLEs, ranging from 16.6 among men with job strain to 18.6 among women without job strain. The proportion of life spent without chronic disease between 50 and 75 ranged from 68.0 among men with job strain to 74.3 among women without job strain (online supplementary table 7).

**Table 3:** Chronic disease-free life expectancy between the ages of 50 and 75 among people without chronic disease at baseline, by job strain, sex and occupational position

	Men		Women	
	n	Chronic disease-free life expectancy (95% CI)*	n	Chronic disease-free life expectancy (95% CI)*
All	25 690		39 142	
No job strain	21 652	17.4 (17.2 to 17.6)	31 603	18.6 (18.4 to 18.8)
Job strain	4038	16.6 (16.2 to 16.9)	7539	18.0 (17.7 to 18.3)
High grade				
No job strain	7840	17.9 (17.5 to 18.2)	6991	19.0 (18.6 to 19.3)
Job strain	1448	16.9 (16.3 to 17.5)	2551	18.4 (17.7 to 19.1)
Middle grade				
No job strain	9086	17.5 (17.2 to 17.8)	17 477	18.8 (18.5 to 19.0)
Job strain	1628	16.7 (16.2 to 17.3)	3774	18.0 (17.6 to 18.4)
Low grade				
No job strain	4726	16.8 (16.4 to 17.1)	7135	18.1 (17.8 to 18.5)
Job strain	962	16.1 (15.6 to 16.7)	1214	17.5 (17.0 to 18.0)

*Estimated life years spend free of chronic disease between ages of 50 and 75.

#### CDFLE additionally stratified by occupational position

Among people without chronic disease at baseline, the CDFLEs were shorter in both men and women and in all occupational grades, and the differences were statistically significant in all groups except among high-grade women (online supplementary table 8, supplementary figure 2). Lower CDFLEs were found in almost all strata in the separate analyses for each cohort, although the patterns were a bit more inconsistent across cohort among people without chronic disease at baseline (online supplementary table 11a,b).

### Sensitivity analysis

Similar differences in HLE and CDFLE, between exposed and unexposed, were observed when job strain was compared with low strain (ie, low demands and high control at work; online supplementary tables 12–13). Overall, men with job strain had 2.5 years and women with job strain 2.4 years shorter HLE when compared with counterparts with low strain. For both men and women, the differences in HLE were most pronounced in low-grade occupations (3 years compared with, eg, 2.3 and 2.1 among middle-grade men and women). Men also had 1.6 years shorter CDFLE, while women had 0.8 years shorter CDFLE.

## Discussion

This multicohort study from four European countries showed that job strain was associated with shorter HLE from age 50 to 75. The association was found within all occupational grades, consistently in all four cohorts and in both sexes, but was most pronounced among men and participants in lower occupational positions. A weaker association was also observed for CDFLE.

There were no marked differences in life expectancies between individuals with and without job strain when accounting for occupational grade. This corresponds with studies from the IPD-Work consortium, in which job strain had a moderate impact on coronary heart disease,^5^ and none on the major cancer types,^22^ which are major causes of death, and from studies on mortality risk.^23–25^

The robust association between job strain and shorter HLE is also consistent with earlier findings of associations between job strain and self-reported health,^26 27^ as well as with studies indicating that retirement from work with job demands is associated with decreased risk of suboptimal self-rated health.^4^ A number of studies have also demonstrated associations between job strain and chronic diseases such as coronary heart disease and stroke,^5 6 28^ and diabetes,^7^ whereas no clear association have been found for cancer,^22^ respiratory diseases such as asthma,^29^ and chronic obstructive pulmonary disease.^30^ Some review studies have also suggested stronger associations among men between job strain and blood pressure^31^ or general stress and diabetes,^32^ which is in line with the results of the present study. However, associations between job strain is generally found in both men and women and more research seems warranted on potential gender differences. In the present study, the association between job strain and HLE also varied by occupational grade and was generally more pronounced in the lower occupational grades. This is also in line with some previous research.^33^ However, only few previous large-scale studies have studied the relationships between work characteristics and health by socioeconomic status.^5 33^ For HLE, there seemed to be a three-way multiplicative interaction in the present study, which indicated that the associations between job strain and healthy/disease-free life expectancy differed by both sex and occupational position on the multiplicative scale. These findings of a more pronounced association in men and lower occupational grades should be confirmed by further studies. In future studies, it would also be of interest to examine whether there are additive interactions, which can clarify if interventions to job strain in specific subgroups are worthwhile from a public health perspective.^34^ The association with HLE/CDFLE varied somewhat between cohorts, which may be explained by differences in cohort characteristics and definitions of job strain, self-rated health and chronic disease. For example, three out of the four cohorts represent specific segments of the labour force rather than being representative of the whole national working population, which may also limit the generalisability of the results. The cohorts also differed somewhat with regard to sex, age and occupational position. Potential differences in the categorisation of occupational position may have contributed to underestimate differences by socioeconomic position, but we believe any differences are likely to have minor influence on the results. The measure of occupational position was also equally related to HLE in SLOSH, FPS and GAZEL in earlier work (Head *et al* manuscript). In line with expectations, those in lower occupational positions had a shorter life expectancy until age 75 and could expect to live fewer of these years in good health. The main variables were also harmonised beforehand. The operationalisations of job strain have been harmonised as part of the IPD-Work consortium and validated.^18^ The definition of job strain differed slightly between GAZEL/SLOSH, FPS and WHII. However, earlier work has shown good correspondence between some alternative job strain operationalisations.^18^ A sensitivity analysis of job strain compared with low strain also supported the main conclusions suggesting that the results were robust to alternative operationalisations. When it comes to the health measures, the measure of chronic disease spanned different time frames between the cohorts, and there were some differences in diseases covered. For example, an assessment of stroke was not separately available in SLOSH. Moreover, cancer data were retrieved from self-reports in some cohorts while register data were used in other cohorts. This may at least partially explain the more pronounced heterogeneity between the cohorts with regard to CDFLE. Similar differences in CDFLE by job strain were, however, observed with different operationalisations of chronic disease suggesting that the results were robust. There were also differences in frequency of follow-up intervals between studies ranging from annual to 4-yearly waves of data collection, but sensitivity analyses in a previous study indicate that this is not a major source of bias (Head *et al* manuscript).

Although there were some differences between the cohorts, the use of data from four cohorts, including a large number of individuals, provided good power to investigate several measures of health expectancy. Major strengths of the study are also the prospective design, long follow-up and high-quality harmonised data. However, there are also some limitations. Participants in cohort studies tend to be healthier than non-participants since those with job strain and ill health may be less likely to participate in the survey, a problem which is exacerbated with prolonged follow-up. Also, drop-out may increase past retirement. This selection mechanism may result in an overestimation of (healthy) life expectancy and an underestimation of the impact of the occupational exposures. On the other hand, there is a risk that residual confounding or common method bias could inflate the association between job strain and health expectancy. Some candidate confounders we did not account for, but may also act as intermediate variables, were health behaviours that are linked to health and disease.^35–37^ Physical occupational exposure has also been related to self-rated health^27^ as well as HLE and CDFLE.^38^

We conclude that while earlier research has generally found significant but relatively modest associations between job strain and a number of individual health outcomes, the present study indicates that the total effect of job strain on health expectancy could be fairly substantial, particularly in regard to health expectancy based on self-rated health and among men in lower occupational positions. This association was not explained by occupational grade, suggesting an influence over and above that of the social gradient. The association of job strain with CDFLE was weaker than for HLE, possibly indicating that the impact is mainly on perceived health. Another possible explanation is that self-rated health is a more all-encompassing measure incorporating both mental and physical health symptoms. In future research, it would be of interest to study health expectancy based on a more comprehensive measure of chronic disease. Intervention studies are, however, needed to investigate whether these associations are indeed causal and not due to selection or residual confounding. To fully understand the extent to which job strain impacts on health expectancy, future studies may also need to follow people from an earlier age and investigate the association with health expectancy beyond the age of 75.

**What is already known about this subject?**

- Job strain has been shown to be associated with an increased risk of various health conditions, including coronary heart disease, stroke, diabetes, musculoskeletal disorders and depression, but the role of job strain in overall physical and mental health is not well understood.
- No previous study has to our knowledge assessed the extent to which job strain is related to health expectancy, which takes both health status and mortality into account.

**What are the new findings?**

- This study suggests that individuals with job strain live fewer years with good self-rated health and fewer years free from chronic disease, although the association with chronic disease-free life expectancy was weaker.
- This is the first study to our knowledge on job strain and health expectancy, which suggests a more substantial public health impact of job strain than previously recognised.

**How might this impact on policy or clinical practice in the foreseeable future?**

- The results suggest that interventions to job strain may contribute to more healthy life years in later life.
