# Supplementary figures and images for: Job strain and loss of healthy life years between ages 50 and 75 by sex and occupational position: analyses of 64 934 individuals from four prospective cohort studies
Source: Occup Environ Med. 2018 May 7;75(7):486–93. doi: 10.1136/oemed-2017-104644 (PMC6035484; doi:10.1136/oemed-2017-104644)

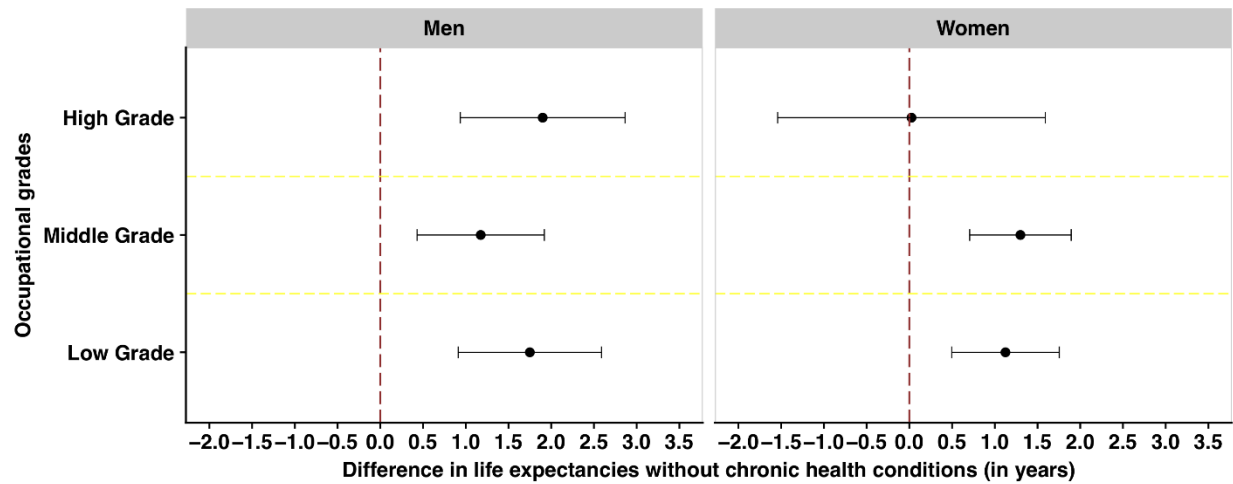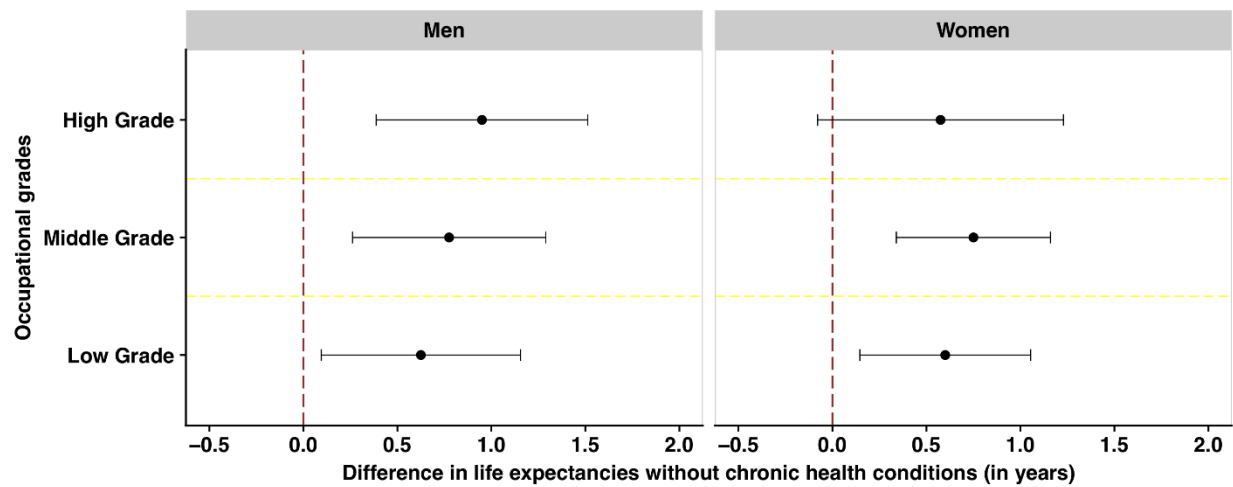

Supplement: Supplementary data [file oemed-2017-104644supp001.pdf]
