# Supplementary material for: Job strain and loss of healthy life years between ages 50 and 75 by sex and occupational position: analyses of 64 934 individuals from four prospective cohort studies
Source: Occup Environ Med. 2018 May 7;75(7):486–93. doi: 10.1136/oemed-2017-104644 (PMC6035484; doi:10.1136/oemed-2017-104644)
Supplement: Supplementary data [file oemed-2017-104644supp002.pdf]

eTable 1. Distribution of follow-up time in years during the study period by age at inclusion.

Age at inclusion	Follow-up time (years)			No. of individuals
	Minimum	Maximum	Mean	
**FPS (Finland)**				
50 – 54	0	16	6.1	25225
55 – 59	0	16	8.5	8909
60 – 64	0	16	8.9	1610
65 – 69	0	9	3.3	36
70 – 74	-	-	-	-
All ages	0	16	6.8	35780
**GAZEL (France)**				
50 – 54	0	17	13.8	10251
55 – 59	0	17	14.2	1040
60 – 64	0	13	1.9	11
65 – 69	-	-	-	-
70 – 74	-	-	-	-
All ages	0	17	13.8	11302
**SLOSH (Sweden)**				
50 – 54	0	8	4.8	3482
55 – 59	0	8	7.3	2016
60 – 64	0	8	7.4	1958
65 – 69	0	8	7.1	850
70 – 74	0	2	0.4	25
All ages	0	8	6.0	8330
**WHII (UK)**				
50 – 54	0	28	15.6	7680
55 – 59	0	28	14.9	1250
60 – 64	0	23	6.0	155
65 – 69	0	16	1.2	27
70 – 74	-	-	-	-
All ages	0	28	15.2	9121

eTable 2. Operationalization of exposure and outcome variables in each study cohort.

	Job strain	Self-rated health*	Chronic disease#
**FPS (Finland)**	Job Content Questionnaire (JCQ)<br><br>**Questions demand scale:**<br>1. Working very hard/intensively<br>2. No excessive amount of work/too much effort<br>3. Enough time<br><br>**Questions control scale:**<br>1. Learn new things<br>2. High level of skill<br>3. Creativity/initiative<br>4. Repetitive work<br>5. A lot of say/what to do<br>6. Little freedom/how to do<br><br>**Job strain:** a combination of mean demands scores above the median and mean control scores below the median.	Question: "How is your health?"<br><br>**Good health**: Good, Fairly good<br>**Suboptimal health**: Average, Fairly poor and Poor	Question: "Has a doctor told you that you have or have had …?<br>(1) myocardial infarction or angina pectoris, (2) stroke, (3) chronic bronchitis or asthma, (4) diabetes or high blood sugar; and (5) cancer or a malignant tumour of any kind except skin cancer*"<br><br>* For the first two waves cancer was not inquired, thus information from cancer register was used<br><br>**No chronic disease**: No to any of the above<br>**Chronic disease**: Yes to any of the above
**GAZEL (France)**	Job Content Questionnaire (JCQ)<br><br>**Questions demands scale:**<br>1. Working fast<br>2. Working hard/intensively<br>3. No excessive amount of work/too much effort<br>4. Enough time<br>5. Conflicting demands	Question: "How would you judge the state of your general health?"<br><br>The respondents were asked to rate their health on an eight level scale ranging from "very good" (coded 1) to "very poor" (coded 8)<br><br>**Good health**: Scores 1-4	Subjects were asked to put a check mark next to the diseases in a list of diseases and symptoms from which they had suffered over the past 12 months including:<br>Myocardial infarction<br>Diabetes<br>Stroke<br>Chronic bronchitis<br>Asthma<br>Cancer

	**Questions control scale:**<br>1. Learn new things<br>2. High level of skill<br>3. Creativity/initiative<br>4. Repetitive work<br>5. A lot of say/what to do<br>6. Little freedom/how to do<br><br>**Job strain:** a combination of mean demands scores above the median and mean control scores below the median.	**Suboptimal health** Scores 5-8	**No chronic disease** Absence of any of the above<br>**Chronic disease** Presence of any of the above
**SLOSH (Sweden)**	Demand Control Questionnaire (DCQ)<br><br>**Questions demands scale:**<br>1. Working fast<br>2. Working hard/intensively<br>3. No excessive amount of work/too much effort<br>4. Enough time<br>5. Conflicting demands<br><br>**Questions control scale:**<br>1. Learn new things<br>2. High level of skill<br>3. Creativity/initiative<br>4. Repetitive work<br>5. A lot of say/what to do<br>6. Little freedom/how to do<br><br>**Job strain:** a combination of mean demands scores above the median and mean control scores below the median.	Question: "How would you rate your general state of health?"<br><br>**Good health** Very good, Quite good<br>**Suboptimal health** Neither good nor bad, Quite poor and Very poor	Question: "Do you have or have you had any of the following long-standing and/or serious diseases or complaints during the past 2 years and how much has it impacted on your life?"<br>(1) cardiovascular disease, (2) chronic obstructive lung disease, (3) asthma and (4) diabetes<br><br>Response options for each condition:<br>No<br>Yes, but it does not impact on my life at all<br>Yes, impacts my life somewhat<br>Yes, impacts my life a lot<br><br>Information on cancer is from cancer register.<br><br>**No chronic disease** No to any of the above and no malignant cancer diagnose in the cancer register<br>**Chronic disease** Yes to any of the above or a malignant

			cancer diagnose in the cancer register
**WHII (UK)**	Minor modification of Job Content Questionnaire (JCQ)<br><br>**Questions demand scale:**<br>1. Working very fast<br>2. Working very hard/intensively<br>3. Enough time<br>4. Conflicting demands<br><br>**Questions control scale:**<br>1. Learn new things<br>2. High level of skill<br>3. Creativity/initiative<br>4. Repetitive work<br>5. A lot of say/what to do<br>6. Little freedom/how to do<br><br>**Job strain:** a combination of mean demands scores above the median and mean control scores below the median.	Question: "In general would you say your health is…?"<br><br>**Good health** — Excellent, Very good, Good<br>**Suboptimal health** — Fair and Poor	Question: "Has a doctor ever told you that you have had …<br>(1) a heart attack or any other heart trouble? (enlarged heart, fluid on lungs, heart failure), (2) stroke (stroke or transient ischaemic attack), (4) Diabetes"<br><br>Question. "Do you have any longstanding illness, diseases or medical conditions of which you have sought treatment in the last 12 months? If yes, please list.<br>(3) chronic lung disease (includes conditions coded as chronic bronchitis or asthma)<br><br>Information on cancer is from cancer register.<br><br>**No chronic disease** — No to any of the above and no cancer diagnose in the cancer register<br>**Chronic disease** — Yes to any of the above or a cancer diagnose in the cancer register

*Used in combination with mortality to assess healthy life-expectancy (HLE)
Used in combination with mortality to assess chronic-disease free life expectancy (CDFLE)

eTable 3. Cohort specific odds ratios for self-reported health transitions from multinomial logistic models.

	Transition			
	Healthy to Unhealthy OR (95% CI)*	Unhealthy to Healthy OR (95% CI)*	Healthy to Death OR (95% CI)*	Unhealthy to Death OR (95% CI)*
**FPS (Finland)**				
No job strain	1.00	1.00	1.00	1.00
Job strain	1.69 (1.63-1.76)	0.59 (0.57-0.62)	1.55 (1.31-1.84)	0.92 (0.77-1.09)
**GAZEL (France)**				
No job strain	1.00	1.00	1.00	1.00
Job strain	1.53 (1.47-1.58)	0.65 (0.63-0.68)	1.16 (0.93-1.45)	0.76 (0.61-0.95)
**SLOSH (Sweden)**				
No job strain	1.00	1.00	1.00	1.00
Job strain	1.48 (1.32-1.67)	0.67 (0.60-0.76)	2.13 (1.04-4.38)	1.44 (0.70-2.96)
**WHII (UK)**				
No job strain	1.00	1.00	1.00	1.00
Job strain	1.42 (1.33-1.51)	0.71 (0.66-0.75)	1.14 (0.98-1.34)	0.81 (0.69-0.95)

*adjusted for age, sex and occupational position.

eTable 4. Cohort specific odds ratios for chronic disease transitions from multinomial logistic models.

	Healthy to Unhealthy OR (95% CI)*	Healthy to Death OR (95% CI)*	Unhealthy to Death OR (95% CI)*
**FPS (Finland)**			
No job strain	1.00	1.00	1.00
Job strain	1.22 (1.18-1.27)	1.16 (0.99-1.35)	0.95 (0.81-1.11)
**GAZEL (France)**			
No job strain	1.00	1.00	1.00
Job strain	1.15 (1.12-1.19)	1.14 (0.91-1.42)	0.99 (0.79-1.24)
**SLOSH (Sweden)**			
No job strain	1.00	1.00	1.00
Job strain	1.24 (1.11-1.39)	2.18 (1.06-4.49)	1.76 (0.86-3.61)
**WHII (UK)**			
No job strain	1.00	1.00	1.00
Job strain	1.15 (1.09-1.21)	1.15 (0.98-1.34)	1.00 (0.85-1.17)

*adjusted for age, sex and occupational position.

eTable 5. Combined partial life expectancy, healthy life expectancy (HLE) and proportion of life spent in good health between the ages of 50 and 75 by job strain and sex

	Life expectancy (95% CI)	Healthy life expectancy (95% CI)	Unhealthy life expectancy (95% CI)	Proportion of life spent in good health (%)
**Men**				
No job strain	24.5 (24.4-24.6)	19.4 (19.2-19.6)	5.1 (5.0-5.3)	78.9 (78.2-79.7)
Job strain	24.1 (23.8-24.4)	17.4 (17.0-17.8)	6.7 (6.4-7.0)	72.0 (70.6-73.4)
**Women**				
No job strain	25.0 (24.9-25.1)	19.7 (19.5-19.9)	5.3 (5.2-5.5)	78.6 (78.0-79.2)
Job strain	24.9 (24.8-25.1)	18.1 (17.9-18.4)	6.8 (6.6-7.1)	72.6 (71.6-73.7)

eTable 6. Combined partial life expectancy, chronic-disease free life expectancy (CDFLE) and proportion of life spent free of chronic disease between the ages of 50 and 75 by job strain and sex, among all individuals with data on chronic disease

	Life expectancy (95% CI)	Chronic disease-free life expectancy (95% CI)	Unhealthy life expectancy (95% CI)	Proportion of life spent free from chronic disease (%)
**Men**				
No job strain	24.5 (24.4-24.6)	13.6 (13.4-13.9)	10.9 (10.7-11.1)	55.6 (54.7-56.5)
Job strain	24.3 (24.2-24.5)	12.0 (11.5-12.5)	12.3 (11.8-12.8)	49.4 (47.3-51.6)
**Women**				
No job strain	25.0 (24.9-25.1)	14.6 (14.4-14.9)	10.4 (10.1-10.6)	58.5 (57.5-59.5)
Job strain	24.9 (24.8-25.0)	13.8 (13.2-14.4)	11.1 (10.5-11.7)	55.4 (52.8-58.0)

eTable 7. Combined partial life expectancy, chronic disease-free life expectancy and proportion of life spent without chronic disease between the ages of 50 and 75 by job strain and sex, among people without chronic disease at baseline

	Life expectancy (95% CI)	Chronic disease- free life expectancy (95% CI)	Life expectancy with chronic disease (95% CI)	Proportion of life spent free from chronic disease (%)
**Men**				
No job strain	24.6 (24.5-24.7)	17.4 (17.2-17.6)	7.2 (7.0-7.4)	70.6 (69.9-71.4)
Job strain	24.4 (24.2-24.6)	16.6 (16.2-16.9)	7.8 (7.5-8.2)	68.0 (66.5-69.5)
**Women**				
No job strain	25.1 (25.0-25.1)	18.6 (18.4-18.8)	6.5 (6.3-6.6)	74.3 (73.5-75.0)
Job strain	25.0 (24.9-25.1)	18.0 (17.7-18.3)	7.1 (6.7-7.4)	71.8 (70.5-73.2)

eTable 8. Combined partial life expectancy, chronic disease-free life expectancy (CDFLE) and proportion of life spent without chronic health conditions between the ages of 50 and 75 by occupational position and job strain for men and women without chronic health disease at baseline.

	Life expectancy (95% CI)	Healthy life expectancy (95%CI)	Unhealthy life expectancy (95%CI)	Proportion of life spent in good health (%)
**Men**				
High grade				
No job strain	25.0 (24.9-25.1)	17.9 (17.5-18.2)	7.2 (6.8-7.5)	71.4 (70.0-72.8)
Job strain	24.9 (24.7-25.1)	16.9 (16.3-17.5)	8.0 (7.4-8.6)	68.0 (65.5-70.4)
Middle grade				
No job strain	24.7 (24.5-24.8)	17.5 (17.2-17.8)	7.1 (6.8-7.5)	71.0 (69.9-72.2)
Job strain	24.4 (24.1-24.8)	16.7 (16.2-17.3)	7.7 (7.1-8.2)	68.5 (66.1-70.9)
Low grade				
No job strain	24.2 (24.0-24.3)	16.8 (16.4-17.1)	7.4 (7.0-7.8)	69.4 (68.0-70.8)
Job strain	23.9 (23.6-24.2)	16.1 (15.6-16.7)	7.8 (7.2-8.3)	67.6 (64.8-70.3)
**Women**				
High grade				
No job strain	25.3 (25.2-25.4)	19.0 (18.6-19.3)	6.4 (6.0-6.7)	74.9 (73.4-76.4)
Job strain	25.4 (25.1-25.6)	18.4 (17.7-19.1)	7.0 (6.3-7.6)	72.6 (69.5-75.6)
Middle grade				
No job strain	25.1 (25.0-25.2)	18.8 (18.5-19.0)	6.3 (6.1-6.6)	74.7 (73.5-75.9)
Job strain	24.9 (24.7-25.1)	18.0 (17.6-18.4)	6.9 (6.5-7.3)	72.3 (70.4-74.1)
Low grade				
No job strain	24.8 (24.6-24.9)	18.1 (17.8-18.5)	6.7 (6.3-7.0)	73.1 (71.9-74.4)
Job strain	24.8 (24.6-25.0)	17.5 (17.0-18.0)	7.3 (6.8-7.7)	70.6 (68.7-72.6)

eTable 9a. Partial life expectancy, healthy life expectancy (HLE) and proportion of life spent in good health between the ages of 50 and 75 by occupational position and job strain for *men*.

	Life expectancy (95% CI)	Healthy life expectancy (95%CI)	Unhealthy life expectancy (95%CI)	Proportion of life spent in good health (%)
**FPS (Finland)**				
High grade				
No job strain	24.7 (24.4-24.9)	19.2 (18.7-19.8)	5.4 (5.0-5.9)	78.0
Job strain	24.4 (23.9-24.9)	16.6 (15.6-17.6)	7.8 (6.9-8.7)	67.9
Middle grade				
No job strain	24.1 (23.7-24.6)	16.4 (15.7-17.0)	7.8 (7.2-8.4)	67.8
Job strain	24.1 (23.5-24.6)	14.0 (13.1-14.9)	10.1 (9.2-10.9)	58.2
Low grade				
No job strain	23.8 (23.3-24.3)	13.3 (12.5-14.1)	10.4 (9.7-11.2)	56.1
Job strain	23.6 (23.0-24.2)	10.1 (9.3-10.9)	13.5 (12.6-14.3)	42.9
**GAZEL (France)**				
High grade				
No job strain	24.9 (24.7-25.0)	21.9 (21.6-22.2)	3.0 (2.8-3.2)	88.0
Job strain	24.8 (24.4-25.2)	20.5 (20.0-21.1)	4.3 (3.8-4.7)	82.9
Middle grade				
No job strain	24.6 (24.4-24.7)	21.0 (20.8-21.3)	3.5 (3.3-3.7)	85.7
Job strain	24.4 (24.1-24.8)	19.4 (18.9-19.9)	5.1 (4.7-5.5)	79.3
Low grade				
No job strain	24.1 (23.7-24.5)	19.6 (19.0-20.1)	4.6 (4.2-4.9)	81.1
Job strain	24.2 (23.6-24.7)	17.6 (16.9-18.3)	6.5 (6.0-7.1)	72.9
**SLOSH (Sweden)**				
High grade				
No job strain	25.1 (24.3-25.8)	19.8 (18.6-21.0)	5.2 (4.2-6.3)	79.1
Job strain	23.9 (21.6-26.2)	18.2 (15.7-20.7)	5.7 (4.0-7.4)	76.1
Middle grade				
No job strain	24.7 (24.1-25.3)	20.5 (19.6-21.5)	4.2 (3.4-4.9)	83.1
Job strain	23.6 (21.6-25.7)	18.3 (16.1-20.6)	5.3 (3.8-6.8)	77.6
Low grade				
No job strain	25.3 (24.7-25.8)	18.8 (17.8-19.8)	6.5 (5.6-7.4)	74.4
Job strain	24.5 (23.5-25.4)	16.6 (14.8-18.4)	7.8 (6.3-9.4)	67.9
**Whitehall II (UK)**				
High grade				
No job strain	24.9 (24.7-25.0)	22.5 (22.2-22.7)	2.4 (2.2-2.6)	90.4
Job strain	24.8 (24.5-25.1)	21.6 (21.0-22.3)	3.2 (2.6-3.8)	87.2
Middle grade				
No job strain	24.4 (24.3-24.6)	21.0 (20.7-21.3)	3.4 (3.2-3.6)	86.1
Job strain	24.4 (24.1-24.7)	20.1 (19.5-20.6)	4.3 (3.8-4.9)	82.3
Low grade				
No job strain	23.6 (23.2-24.0)	18.2 (17.5-19.0)	5.4 (4.8-5.9)	77.3
Job strain	23.1 (22.5-23.7)	15.9 (14.6-17.2)	7.2 (6.0-8.4)	68.7

eTable 9b. Partial life expectancy, healthy life expectancy (HLE) and proportion of life spent in good health between the ages of 50 and 75 by occupational position and job strain for *women*.

	Partial life expectancy between the ages of 50 and 75			
	Life expectancy (95% CI)	Healthy life expectancy (95%CI)	Unhealthy life expectancy (95%CI)	Proportion of life spent in good health (%)
**FPS (Finland)**				
High grade				
No job strain	25.2 (25.0-25.3)	19.5 (19.1-19.8)	5.7 (5.4-6.1)	77.3
Job strain	25.1 (24.8-25.3)	17.1 (16.4-17.7)	8.0 (7.4-8.6)	68.1
Middle grade				
No job strain	25.0 (24.9-25.2)	17.4 (17.1-17.8)	7.6 (7.3-7.9)	69.6
Job strain	24.9 (24.7-25.1)	15.2 (14.8-15.6)	9.7 (9.4-10.1)	60.9
Low grade				
No job strain	24.7 (24.4-25.0)	13.8 (13.0-14.5)	10.9 (10.2-11.7)	55.7
Job strain	24.7 (24.4-24.9)	11.4 (10.8-12.0)	13.3 (12.7-13.8)	46.2
**GAZEL (France)**				
High grade				
No job strain	25.1 (24.9-25.4)	21.7 (21.3-22.1)	3.5 (3.1-3.8)	86.3
Job strain	25.1 (24.6-25.7)	20.8 (19.9-21.7)	4.3 (3.5-5.1)	82.9
Middle grade				
No job strain	25.0 (24.8-25.2)	21.0 (20.7-21.4)	4.0 (3.7-4.2)	84.1
Job strain	25.0 (24.7-25.3)	19.5 (19.0-20.0)	5.5 (5.1-6.0)	77.9
Low grade				
No job strain	24.7 (24.3-25.0)	19.7 (19.2-20.2)	5.0 (4.6-5.4)	79.7
Job strain	24.7 (24.3-25.0)	17.6 (16.9-18.2)	7.1 (6.5-7.6)	71.3
**SLOSH (Sweden)**				
High grade				
No job strain	25.6 (25.3-26.0)	20.7 (19.7-21.7)	4.9 (4.0-5.9)	80.7
Job strain	25.2 (24.5-26.0)	19.8 (18.3-21.3)	5.4 (4.1-6.8)	78.4
Middle grade				
No job strain	25.5 (25.2-25.8)	21.6 (21.0-22.2)	3.9 (3.3-4.5)	84.7
Job strain	25.1 (24.4-25.7)	20.3 (19.1-21.4)	4.8 (3.8-5.8)	80.9
Low grade				
No job strain	25.7 (25.4-26.0)	19.5 (18.5-20.5)	6.2 (5.2-7.1)	76.0
Job strain	25.4 (24.9-25.9)	18.3 (16.9-19.8)	7.1 (5.8-8.4)	72.2
**Whitehall II (UK)**				
High grade				
No job strain	25.1 (24.9-25.3)	22.5 (22.1-23.0)	2.5 (2.2-2.9)	89.8
Job strain	25.3 (24.9-25.6)	22.1 (21.1-23.1)	3.1 (2.2-4.1)	87.5
Middle grade				
No job strain	24.7 (24.5-24.9)	20.9 (20.5-21.4)	3.8 (3.4-4.1)	84.7
Job strain	24.8 (24.5-25.1)	19.5 (18.6-20.3)	5.3 (4.5-6.1)	78.6
Low grade				
No job strain	23.9 (23.7-24.2)	17.9 (17.4-18.4)	6.1 (5.6-6.6)	74.6
Job strain	24.2 (23.7-24.6)	16.1 (15.1-17.1)	8.1 (7.1-9.0)	66.6

eTable 10a. Partial life expectancy, chronic disease-free life expectancy (CDFLE) and proportion of life spent without chronic health conditions between the ages of 50 and 75 by occupational position and job strain for men with data on chronic disease

	Life expectancy (95% CI)	Chronic disease-free life expectancy (95%CI)	Life expectancy with chronic disease (95%CI)	Proportion of life spent without chronic disease (%)
**FPS (Finland)**				
High grade				
No job strain	24.5 (24.1-24.7)	14.7 (13.9-15.0)	9.7 (9.4-10.6)	60.2
Job strain	24.4 (23.9-24.8)	13.3 (11.6-14.6)	11.1 (9.7-12.7)	54.4
Middle grade				
No job strain	24.1 (23.6-24.3)	13.3 (12.4-13.9)	10.9 (10.1-11.5)	55.0
Job strain	24.1 (23.2-24.5)	11.2 (9.5-13.0)	13.0 (10.9-14.4)	46.3
Low grade				
No job strain	23.5 (23.0-23.8)	12.7 (12.0-13.1)	10.8 (10.3-11.5)	54.0
Job strain	23.1 (22.6-23.9)	9.9 (9.2-11.2)	13.2 (12.0-14.3)	42.9
**GAZEL (France)**				
High grade				
No job strain	24.8 (24.7-25.0)	15.3 (14.9-15.7)	9.5 (9.1-10.0)	61.7
Job strain	24.8 (24.4-25.1)	14.1 (13.0-15.8)	10.6 (9.0-11.8)	57.1
Middle grade				
No job strain	24.5 (24.4-24.7)	14.5 (14.2-14.8)	10.0 (9.7-10.4)	59.1
Job strain	24.5 (24.1-24.8)	14.3 (13.2-14.8)	10.2 (9.7-11.4)	58.5
Low grade				
No job strain	24.0 (23.7-24.4)	13.4 (12.8-14.3)	10.6 (9.9-11.4)	55.9
Job strain	24.1 (23.6-24.6)	13.7 (12.3-14.7)	10.4 (9.4-11.9)	56.7
**SLOSH (Sweden)**				
High grade				
No job strain	25.7 (25.4-25.9)	13.9 (12.7-15.3)	11.7 (10.3-12.9)	54.3
Job strain	25.5 (24.6-25.9)	9.6 (6.5-12.4)	15.9 (13.3-19.2)	37.6
Middle grade				
No job strain	25.2 (24.8-25.5)	13.5 (12.4-14.9)	11.6 (10.4-12.8)	53.8
Job strain	24.3 (23.2-25.4)	11.7 (9.7-13.9)	12.5 (10.7-14.8)	48.4
Low grade				
No job strain	25.4 (25.0-25.7)	12.8 (11.7-13.9)	12.6 (11.5-13.8)	50.4
Job strain	24.7 (24.1-25.5)	11.3 (9.8-13.1)	13.4 (11.9-15.0)	45.7
**Whitehall II (UK)**				
High grade				
No job strain	24.8 (24.7-25.0)	13.2 (12.6-13.5)	11.7 (11.3-12.2)	53.1
Job strain	24.7 (24.6-25.1)	12.6 (10.9-13.6)	12.1 (11.2-13.9)	51.0
Middle grade				
No job strain	24.4 (24.2-24.5)	12.9 (12.5-13.4)	11.5 (11.0-12.0)	53.0
Job strain	24.4 (24.1-24.7)	12.3 (11.5-13.3)	12.1 (11.1-12.9)	50.5
Low grade				
No job strain	23.4 (23.0-23.8)	13.3 (12.3-14.1)	10.1 (9.4-11.2)	56.7
Job strain	23.3 (22.6-24.1)	10.3 (7.4-12.0)	13.0 (11.6-16.1)	44.3

eTable 10b. Partial life expectancy, chronic disease-free expectancy (CDFLE) and proportion of life spent without chronic health conditions between the ages of 50 and 75 by occupational position and job strain for women with data on chronic disease.

	Life expectancy (95% CI)	Chronic disease-free life expectancy (95%CI)	Life expectancy with chronic disease (95%CI)	Proportion of life spent without chronic disease (%)
**FPS (Finland)**				
High grade				
No job strain	25.1 (25.0-25.3)	14.9 (14.6-15.4)	10.2 (9.8-10.6)	59.3
Job strain	25.2 (24.8-25.3)	14.5 (13.1-15.4)	10.7 (9.7-12.0)	57.5
Middle grade				
No job strain	24.9 (24.8-25.0)	15.0 (14.7-15.3)	9.9 (9.6-10.3)	60.2
Job strain	24.8 (24.7-25.1)	13.8 (13.2-14.3)	11.0 (10.5-11.6)	55.8
Low grade				
No job strain	24.6 (24.4-24.8)	14.0 (13.4-14.4)	10.6 (10.1-11.2)	56.9
Job strain	24.6 (24.2-24.8)	12.9 (12.2-13.7)	11.7 (10.8-12.3)	52.4
**GAZEL (France)**				
High grade				
No job strain	25.2 (24.9-25.4)	15.0 (14.1-16.3)	10.2 (9.0-11.1)	59.6
Job strain	25.1 (24.6-25.7)	16.7 (11.3-19.4)	8.4 (6.0-13.7)	66.5
Middle grade				
No job strain	25.0 (24.8-25.2)	15.3 (14.7-15.9)	9.7 (9.2-10.3)	61.0
Job strain	25.0 (24.6-25.2)	14.6 (13.4-15.4)	10.4(9.6-11.6)	58.3
Low grade				
No job strain	24.8 (24.3-25.0)	14.7 (14.1-16.1)	10.1 (8.7-10.6)	59.4
Job strain	24.8 (24.2-25.0)	13.1 (12.1-14.3)	11.6 (10.2-12.6)	53.0
**SLOSH (Sweden)**				
High grade				
No job strain	25.8 (25.6-25.9)	15.5 (14.2-17.0)	10.3 (8.8-11.6)	60.1
Job strain	25.7 (25.1-26.0)	13.4 (10.3-16.8)	12.2 (8.7-15.2)	52.3
Middle grade				
No job strain	25.5 (25.3-25.7)	15.9 (14.6-16.9)	9.6 (8.7-10.7)	62.2
Job strain	24.8 (24.1-25.5)	13.8 (12.3-15.5)	11.0 (9.5-12.8)	55.6
Low grade				
No job strain	25.6 (25.2-25.8)	14.3 (13.4-15.7)	11.3 (9.9-12.1)	55.8
Job strain	25.3 (24.5-25.6)	13.7 (12.2-15.0)	11.6 (10.2-13.0)	54.1
**Whitehall II (UK)**				
High grade				
No job strain	25.0 (24.8-25.2)	12.6 (11.2-13.6)	12.4 (11.4-13.9)	50.4
Job strain	25.1 (24.6-25.5)	13.2 (9.5-15.7)	11.9 (9.5-15.5)	52.5
Middle grade				
No job strain	24.7 (24.5-24.9)	14.0 (13.3-14.9)	10.6 (9.8-11.4)	57.0
Job strain	24.7 (24.4-25.0)	12.8 (11.7-14.3)	11.9 (10.4-12.9)	52.0
Low grade				
No job strain	24.0 (23.7-24.2)	14.4 (13.7-15.0)	9.6 (9.0-10.3)	60.1
Job strain	24.0 (23.6-24.4)	13.2 (11.9-14.6)	10.8 (9.5-12.0)	55.0

eTable 11a. Partial life expectancy, chronic disease-free life expectancy (CDFLE) and proportion of life spent without chronic health conditions between the ages of 50 and 75 by occupational position and job strain for men without chronic health conditions at baseline.

	Life expectancy (95% CI)	Chronic disease-free life expectancy (95%CI)	Life expectancy with chronic disease (95%CI)	Proportion of life spent without chronic disease (%)
**FPS (Finland)**				
High grade				
No job strain	24.6 (24.3-24.8)	18.0 (17.5-18.5)	6.6 (6.1-7.1)	73.3
Job strain	24.4 (23.9-24.9)	17.5 (16.3-18.6)	6.9 (5.8-8.0)	71.7
Middle grade				
No job strain	24.2 (23.9-24.6)	17.6 (17.0-18.2)	6.7 (6.1-7.2)	72.5
Job strain	24.2 (23.5-24.9)	16.7 (15.5-17.9)	7.4 (6.3-8.6)	69.2
Low grade				
No job strain	23.5 (23.2-23.9)	16.0 (15.4-16.5)	7.6 (7.0-8.1)	67.9
Job strain	23.0 (22.4-23.7)	15.4 (14.5-16.2)	7.7 (6.8-8.5)	66.6
**GAZEL (France)**				
High grade				
No job strain	25.0 (24.8-25.1)	18.6 (18.2-19.1)	6.3 (5.9-6.7)	74.7
Job strain	24.9 (24.5-25.3)	18.2 (17.4-19.1)	6.6 (5.8-7.4)	73.3
Middle grade				
No job strain	24.7 (24.6-24.9)	18.3 (18.0-18.6)	6.4 (6.1-6.7)	74.1
Job strain	24.7 (24.4-25.0)	18.2 (17.6 -18.9)	6.5 (5.8-7.1)	73.7
Low grade				
No job strain	24.3 (23.9-24.6)	17.6 (16.9-18.3)	6.7 (6.0-7.3)	72.5
Job strain	24.3 (23.8-24.8)	17.7 (16.8-18.5)	6.6 (5.7-7.5)	72.7
**SLOSH (Sweden)**				
High grade				
No job strain	25.6 (25.3-25.9)	17.1 (16.0-18.2)	8.5 (7.3-9.6)	66.7
Job strain	25.6 (24.7-25.9)	15.0 (13.3-16.9)	10.6 (8.5-12.1)	58.6
Middle grade				
No job strain	25.2 (24.7-25.5)	16.8 (15.8-17.9)	8.4 (7.3-9.2)	66.7
Job strain	24.4 (23.3-25.4)	15.1 (13.3-16.6)	9.3 (7.9-11.0)	61.8
Low grade				
No job strain	25.3 (24.9-25.7)	16.5 (15.5-17.6)	8.8 (7.7-9.8)	65.2
Job strain	24.7 (24.1-25.5)	14.5 (13.3-16.1)	10.2 (8.8-11.6)	58.6
**Whitehall II (UK)**				
High grade				
No job strain	24.9 (24.8-25.1)	17.7 (17.2-18.1)	7.2 (6.8-7.7)	70.9
Job strain	24.7 (24.5-25.0)	16.9 (16.0-17.8)	7.8 (7.0-8.7)	68.3
Middle grade				
No job strain	24.5 (24.3-24.6)	17.4 (17.0-17.7)	7.1 (6.8-7.5)	70.9
Job strain	24.4 (24.2-24.7)	16.9 (16.1-17.7)	7.5 (6.8-8.3)	69.1
Low grade				
No job strain	23.5 (23.1-23.9)	17.0 (16.3-17.7)	6.5 (5.9-7.2)	72.2
Job strain	23.5 (22.7-24.3)	17.0 (15.7-18.3)	6.5 (5.2-7.8)	72.3

eTable 11b. Partial life expectancy, chronic disease-free life expectancy (CDFLE) and proportion of life spent without chronic health conditions between the ages of 50 and 75 by occupational position and job strain for women without chronic health conditions at baseline.

	Life expectancy (95% CI)	Chronic disease-free life expectancy (95%CI)	Life expectancy with chronic disease (95%CI)	Proportion of life spent without chronic disease (%)
**FPS (Finland)**				
High grade				
No job strain	25.1 (25.0-25.2)	19.2 (18.9-19.5)	5.9 (5.6-6.2)	76.5
Job strain	25.2 (24.9-25.4)	18.9 (18.2-19.7)	6.3 (5.5-7.0)	75.1
Middle grade				
No job strain	24.9 (24.8-25.0)	19.0 (18.7-19.2)	5.9 (5.7-6.2)	76.1
Job strain	24.8 (24.5-25.0)	18.3 (17.8-18.8)	6.5 (6.0-6.9)	73.9
Low grade				
No job strain	24.5 (24.3-24.8)	17.7 (17.3-18.2)	6.8 (6.4-7.3)	72.3
Job strain	24.6 (24.3-24.9)	17.1 (16.5-17.8)	7.5 (6.8-8.1)	69.7
**GAZEL (France)**				
High grade				
No job strain	25.3 (24.7-25.9)	19.8 (18.1-21.4)	5.5 (3.9-7.1)	78.1
Job strain	25.4 (25.2-25.6)	19.7 (19.0-20.4)	5.7 (5.0-6.4)	77.6
Middle grade				
No job strain	25.1 (24.8-25.4)	19.2 (18.4-19.9)	5.9 (5.2-6.6)	76.5
Job strain	25.2 (25.0-25.4)	19.2 (18.6-19.7)	6.0 (5.5-6.5)	76.1
Low grade				
No job strain	25.1 (24.7-25.5)	18.6 (17.7-19.5)	6.5 (5.6-7.3)	74.3
Job strain	24.9 (24.6-25.2)	18.4 (17.7-19.2)	6.5 (5.8-7.2)	74.0
**SLOSH (Sweden)**				
High grade				
No job strain	25.7 (25.5-25.9)	19.1 (18.1-20.2)	6.6 (5.5-7.7)	74.3
Job strain	25.6 (25.1-26.0)	17.6 (15.8-19.0)	8.1 (6.5-9.8)	68.5
Middle grade				
No job strain	25.5 (25.3-25.7)	19.0 (18.1-19.7)	6.5 (5.6-7.5)	74.5
Job strain	25.0 (24.4-25.5)	17.3 (16.1-18.5)	7.7 (6.5-9.1)	69.2
Low grade				
No job strain	25.6 (25.3-25.8)	18.6 (17.8-19.6)	7.0 (6.0-7.8)	72.6
Job strain	25.4 (24.8-25.6)	17.1 (15.7-18.1)	8.3 (7.2-9.5)	67.4
**Whitehall II (UK)**				
High grade				
No job strain	25.1 (24.9-25.3)	17.8 (17.1-18.5)	7.3 (6.5-8.0)	71.0
Job strain	25.4 (25.0-25.8)	17.4 (15.9-18.8)	8.0 (6.7-9.3)	68.5
Middle grade				
No job strain	24.8 (24.6-25.0)	17.9 (17.3-18.4)	6.9 (6.4-7.5)	72.1
Job strain	24.8 (24.5-25.1)	17.2 (16.4-18.0)	7.6 (6.8-8.4)	69.4
Low grade				
No job strain	24.1 (23.8-24.3)	17.7 (17.1-18.3)	6.3 (5.8-6.9)	73.6
Job strain	24.1 (23.7-24.6)	17.2 (16.2-18.2)	6.9 (6.0-7.9)	71.2

eTable 12. Healthy life expectancy (HLE) between the ages of 50 and 75 by job strain, sex and occupational position

	Men		Women	
	n	Healthy life expectancy (95% CI)[a]	n	Healthy life expectancy (95% CI)[a]
**All**	11 540		18 594	
Low strain	7 492	19.8 (19.6-20.0)	11 018	20.0 (19.8-20.3)
Job strain	4 048	17.3 (16.9-17.6)	7 576	17.6 (17.3-17.9)
**High grade**				
Low strain	2 560	21.5 (21.2-21.8)	1 591	21.6 (21.2-21.9)
Job strain	1 448	19.2 (18.6-19.7)	2 553	19.5 (18.9-20.1)
**Middle grade**				
Low strain	3 487	19.9 (19.6-20.3)	6 648	20.3 (20.0-20.6)
Job strain	1 629	17.7 (17.2-18.2)	3 785	17.9 (17.5-18.3)
**Low grade**				
Low strain	1 445	18.0 (17.5-18.5)	2 779	18.3 (17.8-18.7)
Job strain	971	15.0 (14.4-15.6)	1 238	15.3 (14.8-15.8)

[a] estimated life years spent in good self-rated health between ages of 50 and 75

eTable 13. Chronic disease-free life expectancy (CDFLE) between the ages of 50 and 75 among people without chronic disease at baseline, by job strain, sex and occupational position

	Men		Women	
	n	Chronic disease-free life expectancy (95% CI)[a]	n	Chronic disease-free life expectancy (95% CI)[a]
**All**	11 519		18 552	
Low strain	7 481	13.6 (13.4-13.9)	11 013	14.6 (14.4-14.9)
Job strain	4 038	12.0 (11.5-12.5)	7 539	13.8 (13.2-14.4)
**High grade**				
Low strain	2 562	16.5 (16.0-17.0)	1 590	16.9 (16.3-17.5)
Job strain	1 448	15.4 (14.7-16.2)	2 551	15.9 (15.2-16.7)
**Middle grade**				
Low strain	3 483	16.3 (15.8-16.8)	6 643	16.7 (16.2-17.2)
Job strain	1 628	15.3 (14.7-15.9)	3 774	15.8 (15.3-16.4)
**Low grade**				
Low strain	1 436	15.4 (14.7-16.1)	2 780	15.9 (15.2-16.6)
Job strain	962	14.2 (13.4-14.9)	1 214	14.9 (14.3-15.6)

[a] estimated life years spent free of chronic disease between ages of 50 and 75
